# Performance Study of a Zirconia-Doped Fiber for Distributed Temperature Sensing by OFDR at 800 °C

**DOI:** 10.3390/s21113788

**Published:** 2021-05-30

**Authors:** Patrick Bulot, Rémy Bernard, Monika Cieslikiewicz-Bouet, Guillaume Laffont, Marc Douay

**Affiliations:** 1 CEA, LIST, Laboratoire Capteurs Fibres Optiques, F-91191 Gif-sur-Yvette, France; guillaume.laffont@cea.fr; 2 UMR 8523-PhLAM-Physique des Lasers Atomes et Molécules, Univ. Lille, F-59000 Lille, France; monika.bouet@univ-lille.fr (M.C.-B.); marc.douay@univ-lille.fr (M.D.)

**Keywords:** distributed optical fiber sensors, OFDR sensing, Rayleigh backscattering, zirconia-doped optical fiber, high temperature sensing

## Abstract

Optical Frequency Domain Reflectometry (OFDR) is used to make temperature distributed sensing measurements along a fiber by exploiting Rayleigh backscattering. This technique presents high spatial and high temperature resolutions on temperature ranges of several hundred of degrees Celsius. With standard telecommunications fibers, measurement errors coming from the correlation between a high temperature Rayleigh trace and the one taken as a reference at room temperature could be present at extremely high temperatures. These correlation errors, due to low backscattering signal amplitude and unstable backscattering signal, induce temperature measurement errors. Thus, for high temperature measurement ranges and at extremely high temperatures (e.g., at 800 °C), a known solution is to use fibers with femtosecond laser inscribed nanograting. These fs-laser-insolated fibers have a high amplitude and thermally stable scattering signal, and they exhibit lower correlation errors. In this article, temperature sensing at 800 °C is reported by using an annealed zirconia-doped optical fiber with an initial 40.5-dB enhanced scattering signal. The zirconia-doped fiber presents initially OFDR losses of 2.8 dB/m and low OFDR signal drift at 800 °C. The ZrO_2_-doped fiber is an alternative to nanograting-inscribed fiber to make OFDR distributed fiber sensing on several meters with gauge lengths of 1 cm at high temperatures.

## 1. Introduction

Optical fibers are used to make sensing measurements in severe environments, especially at high temperature, due to their different interesting properties: they are compact and insensitive to electromagnetic interference; moreover, several sensing techniques allow measurements at several positions along the same fiber [1]. Laser-inscribed fiber Bragg gratings allow quasi-distributed temperature sensing [2,3]. Several techniques exploit light backscattering (Brillouin, Raman or Rayleigh scatterings) to make distributed temperature sensing along the studied optical fiber. These distributed sensing techniques have different spatial resolutions and sensitivities [4].

Optical Frequency Domain Reflectometry (OFDR) is a sensing technique that measures Rayleigh backscattering amplitude along an optical fiber [5,6]. The target of OFDR sensing is to make precise distributed temperature and strain measurements for non-destructive testing, mapping sensing, health monitoring or process optimization with a good spatial resolution along small structures from 10 cm to several tens of meters. Examples of applications at high temperatures are solid oxide fuel cell, steel industry, nuclear plant, aircraft, etc. [7,8,9,10]. OFDR distributed fiber sensors can be deployed on tested structures by embedding, by insertion or by direct contact. They are particularly interesting for embedding applications, due to their small diameter avoiding invasive single-point sensors (like thermocouples) [7,11]. Their reliability is good under 700 °C and it is promising at higher temperatures, especially with specific fibers [8,11,12].

For OFDR temperature distributed sensing, tested optical fiber is used as a sensor and it is put along positions where temperature is to be measured. The OFRD sensing technique is described by Gifford et al. and can be detailed by the following steps [13]. Two OFDR measurements are performed along the tested fiber; the reference measurement conducted at a known temperature and the second measurement conducted at the temperature to be characterized. This fiber is considered as a series of fiber segments, called gauges. For each individual gauge, the backscattering signals of both measurements at the position of the gauge are Fourier transformed back into the frequency domain. The wavelength spectrum of the second measurement at the position of the gauge is compared to the reference by cross-correlation to measure a frequency shift. Indeed, when the temperature at the gauge position changes, the Rayleigh backscattering signal of the gauge is shifted in frequency. This shift can be converted to a temperature variation after calibration of the frequency shift over the operating temperature range. On standard telecommunication fibers (for example SMF-28 fiber from Corning), a temperature resolution of 0.1 °C with gauge length of 1 cm can be reached on a fiber length between 20 and 100 m with commercially available instruments [13,14]. It was experimentally demonstrated that this temperature measurement technique can be used at cryogenic temperatures (for example at −140 °C) or at elevated temperatures but only during short-timed tests (for example: above 800 °C for three minutes) with a pristine telecommunications optical fiber and a room-temperature reference measurement [9,10,11,13,15]. However measurements along telecommunications optical fibers can have correlation errors, especially when the temperature variation is too high over long periods of time (for example: there are errors at ~800 °C during tests of Buric et al. on unmodified SMF-28 fiber with a reference measured at room temperature) [9]. According to Buric et al., these errors could be explained by two means [9,14]. The first explanation is that the low backscattering amplitude of these fibers induces low correlation accuracy: it is difficult to compare two low amplitude backscattering signals. The correlation accuracy gets worse when the frequency shift is too high due to high temperature variation. The second explanation is that physical and chemical changes in the fiber at high temperature modify permanently the Rayleigh scattering profile (these changes are confirmed by annealing tests on fibers at 900 °C [9]), decreasing the similarity of a OFDR measurement compared to the reference one.

When OFDR signal amplitude is improved by UV irradiation, a better temperature resolution is achieved compared to low-backscattering amplitude fibers [14]: an increase of the amplitude improves cross-correlation accuracy. Moreover, if the enhanced Rayleigh backscattering signal remains stable at measured temperatures, then stable temperature measurements are possible above 700 °C [7,9,12]. Several techniques have been studied to improve the OFDR signal amplitude in optical fibers compared to standard communication fibers (like SMF-28 fiber from Corning). The first solution is to increase the numerical aperture of the fiber by changing the chemical doping concentrations of fiber’s core (which increases the refractive index of the core glass compared to that of the cladding). A higher numerical aperture allows to “capture” and guide more Rayleigh scattered light along the fiber, and thus the OFDR signal amplitude is increased. Wegmuller et al. use OFDR to measure the distributed gain along active Er-doped fibers with different concentrations of erbium [16]. Doping with erbium enhances the OFDR signal at the expense of very high losses: a 300 ppm Er-doped fibers has a 3-dB OFDR signal enhancement, but also OFDR signal losses of 2.5 dB/m (due to erbium ion absorption at 1550 nm) [16]. High-level germanium doping gives fibers with higher signal amplitude without heavy losses: the UVS-EPS fiber from CorActive has a 10 dB enhancement compared to SMF-28 fiber [14]. OFDR signal enhancement may also be achieved by a UV laser insolation (uniform or heterogeneous) along a photosensitive optical fiber’s core, through defects, local increase of refractive index or light “reflection” by a fiber Bragg grating at the OFDR wavelength [14,17,18,19]. Defects created by fast neutron irradiations of a sapphire fiber improve also the OFDR signal of the irradiated sapphire fiber, but the signal enhancement disappears at around 300 °C [20]. Another solution consists in creating heat resistant nanograting by a uniform femtosecond laser irradiation scan of a silica glass core fiber [7,9,12,21]. In the study of Yan et al., with this solution, a 17 cm long modified fiber section can have a 40-dB enhancement of Rayleigh backscattering signal [7]. Losses are 15 dB/m without treatment or 10 dB/m after 10 min treatment of the modified fiber in a 10%-hydrogen atmosphere at 800 °C [7]. A more recent study by Wang et al. demonstrates an optimized nanograting-based distributed fiber sensor with losses of 1 dB/m [12]. Particles of small size, compared to light wavelength, induce light scattering in a transparent solid, especially when both materials have different refractive index [22]. Thus, Mg-based oxide nanoparticles or Ca-based nanoparticles induce an increase of the Rayleigh backscattering amplitude in the nanoparticle-doped optical fiber [23,24,25]. The OFDR signal of a Ca-based nanoparticle doped fiber can present an enhancement between 25.9 and 44.9 dB and losses between 0.1 and 8.7 dB/m [24]. Sypabekova et al. demonstrate that this enhancement could be exploited to measure refractive index of an external medium by using OFDR along a Mg-based nanoparticle-doped fiber etched to the core [25].

Several studies assess the temperature measurement performances of various fibers at high temperatures by using OFDR. Chamorovskiy et al. and Popov et al. demonstrate the possibility to make OFDR measurements at 600 °C on aluminum-coated fibers with arrays of fiber Bragg gratings [26,27] (the Rayleigh signal enhancement along the heated fiber Bragg gratings is 30 dB). In studies of Yan et al. and Wang et al., thermally treated nanograting-based distributed fiber sensor allow a stable temperature measurement along the fiber with the heat-treated nanograting at 600 °C in a nuclear reactor, at 800 °C in a solid oxide fuel cell, or even at 1000 °C for optimized fibers [7,12,28].

In this article, we present a zirconia-doped fiber as a new solution to improve OFDR sensing capabilities at elevated temperatures, with an improved OFDR signal amplitude without laser irradiation. Distributed temperature sensing is performed by OFDR with 1 cm-long gauges over a 40 cm-long optical fiber, drawn from a zirconia-doped silica glass optical preform. In the following, this fiber is called ZrO_2_-doped fiber. The zirconia-doped preform is produced by modified chemical vapor deposition (MCVD) method coupled with “solution doping technique” [29]. The preform is chemically characterized by X-ray diffraction and chemical electron microprobe, before drawing to fiber. To compare characteristics of ZrO_2_-doped fiber and SMF-28 fiber, refractive index measurement and OFDR measurements are realized at room temperature. SMF-28 fiber is used as a fiber performance reference for OFDR temperature measurements up to 800 °C. After an annealing step to stabilize physical and chemical properties of ZrO_2_-doped optical fiber, performances of tested ZrO_2_-doped fiber are better than the ones of SMF-28 fiber. This ZrO_2_-doped fiber is a promising alternative to nanograting-inscribed fiber for stable distributed sensing applications at around 800 °C.

## 2. Fiber Fabrication and Characterizations at Room Temperature

### 2.1. Fabrication and Characterization of Zirconia-Doped Optical Fiber

#### 2.1.1. Fabrication of the Zirconia-Doped Optical Fiber

The studied single-mode zirconia-doped fiber is drawn from a preform manufactured in the FiberTech Lille (Lille, France) facilities by MCVD method coupled with “solution doping technique” [29]. Firstly, amorphous and porous silica layers are deposited by MCVD inside a silica glass tube (Heraeus, F300). These layers are doped by germanium and phosphorus to obtain a fiber which can guide light.

Then the soaking solution is prepared from a zirconia sol, according to the procedure described by Le Rouge et al. and Pastre et al. in [30,31]. In addition to this zirconia sol, a silica sol is added by using 7.8 mL of tetraethyl orthosilicate (99.9%, Sigma Aldrich^®^), 6.3 mL of water (µ ohm) and 21.4 mL ethanol (absolute, anhydrous, Sigma Aldrich) as a solvent. After a 1 h soaking step, the solution is removed from the tube and the porous layer is dried at room temperature under controlled atmosphere. The tube with the soaked porous is placed on the MCVD lathe and is consecutively heated to densify and to collapse the tube. The obtained cylinder is then sleeved in another silica glass tube to decrease the ratio between core and cladding diameters on the final preform.

The ZrO_2_-doped fiber is drawn from the preform and is coated with a standard polymer coating. Drawn fiber has a core diameter of 4 µm and a cladding diameter of 105 µm. After test, it has a single-mode behavior between 400 and 1750 nm. A multi-mode behavior is undesired at OFDR wavelength range (from 1530 to 1616 nm) since it prevents using the fiber for OFDR measurements.

#### 2.1.2. Physical, Chemical and Optical Characterizations of the Preform and the Optical Fiber

Chemical Electron Microprobe Analysis (EMPA) is performed across the preform core on a transverse slice. The average molar concentrations of GeO_2_, P_2_O_5_ and ZrO_2_ in the silica glass core are respectively 0.3, 0.4 and 1.9 mol%.

**Figure 1:**
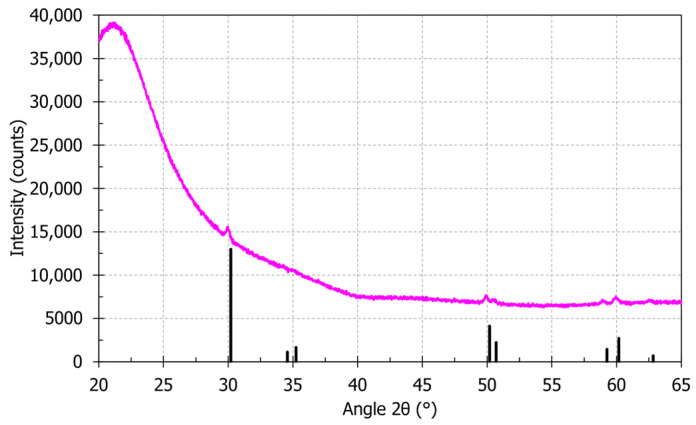
XRD pattern of the preform core and main XRD relative intensity of tetragonal zirconia according to JCPDS reference 01-072-7115.

A slice of the preform is also analyzed by X-ray diffraction (XRD) with a Bruker AXS D8 diffractometer with a Cu Kα radiation source (40 kV and 40 mA) and a Linxeye XE detector (Lille, France). EVA software program is used for phase analyses. Figure 1 shows the XRD pattern of the core of the preform. The pattern has a broad peak at around 21°, which can be explained by amorphous silica glass [32]. Defined peaks of tetragonal phase zirconia are also visible on the XRD pattern, according to JCPDS reference 01-072-7115 [33].

Zirconia comes from the soaking solution. In the soaked porous silica layers, the deposited zirconia gel is initially amorphous. During vitrification and collapsing of the preform, heat treatments are above 1700 °C and induce crystallization of zirconia in tetragonal phase. Zirconia metastable tetragonal phase formation instead of zirconia monoclinic phase (stable phase under 1167 °C for zirconia bulk material) can be explained by the lower surface energy of the tetragonal phase compared to the one of monoclinic phase [34,35]. The tetragonal phase does not change in monoclinic phase after preform heat treatments. This absence of phase change during preform fabrication could be explained by stresses applied on the zirconia entities by the silica glass matrix. The zirconia tetragonal phase is also observed in the works of Brasse et al. on a 30 mol% ZrO_2_-doped silica glass preform core made by solution doping technique [36]. Crystallized zirconia state in the presented preform could be clusters, nanoparticles or particles mixed in the silica glass. Conservation of crystallized zirconia entities in the drawn fiber is possible due to high melting temperature of zirconia (~2680 °C [37]) compared to drawing temperature (<2100 °C). During OFDR tests, zirconia entities should have a significant impact on Rayleigh backscattering inside the fiber, by being a density fluctuation inside the silica glass core.

According to the refractive index profile measured with the IFA-100 device from Interfiber Analysis at 960 nm, the refractive index difference between SMF-28 fiber’s core and cladding is 5.1 × 10^−3^ in average. Core-cladding refractive index difference for the ZrO_2_-doped fiber has been measured to be 8.1 × 10^−3^ in average. Brückner demonstrated that refractive index of some doped silica glasses (for example, doped by germanium or phosphorus) are linearly proportional to the molar concentration of dopants (e.g., GeO_2_ or P_2_O_5_) for wavelengths between 900 and 1700 nm [38]. We consider that it is also the case for glass doped by a low content of ZrO_2_. Thus, measured refractive index differences at 960 nm remain unchanged for wavelengths between 900 and 1700 nm.

Measured values with the IFA-100 device are used to calculate group index in SMF-28 and in ZrO_2_-doped fibers at 1573 nm (median wavelength of used OFDR wavelength range: from 1530 to 1616 nm) according to Equation (A6) defined in Appendix A. For SMF-28 and ZrO_2_-doped fibers, calculated group delays at 1573 nm are respectively 1.4679 and 1.4709. Group delay values for each fiber are used by the OBR software to change OFDR signals from as a function of time to as a function of fiber length.

The cutback method was used to measured optical losses of ZrO_2_-doped fiber: attenuation in transmission is 2 dB/m at 1573 nm. Between 1200 and 1750 nm, attenuation follows a decreasing trend proportional to *λ*^−5^. This level of attenuation allows transmission of light on several meters without problem for OFDR sensing application.

### 2.2. OFDR Measurements at Room Temperature on SMF-28 Fiber and ZrO_2_-Doped FIBER

#### 2.2.1. OFDR Measurement Parameters

A Luna OBR-4600 commercial OFDR interrogator (Gif-sur-Yvette, France) is used to make OFDR measurements along SMF-28 and the ZrO_2_-doped fibers. The software of the OBR-4600 interrogator is used to calculate cross-correlations on OFDR measurements compared to reference at room temperature. The obtained spectral shifts are converted to temperature variations with adapted coefficients of a 6th-degree polynomial. These coefficients are calculated from thermocouple temperature measurements and OFDR spectral shift measurements at the last cooling of following temperature sensing performance tests (between ~22 and ~800 °C). Each fiber has its own coefficients.

OFDR measurement conditions with the interrogator are: wavelength scan from 1530 to 1616 nm; distance range of 30 m; gain 6 dB. In these conditions, according to [39], spatial resolution is around 10 µm for a SMF-28 fiber. For cross-correlation, the “gauge width” is equal to 1 cm (studied fiber length for each gauge), which allows a temperature resolution of 0.1 °C on a SMF-28 fiber according to interrogator performance specifications. The chosen distance between two successive gauges along the fiber is 0.06 cm.

#### 2.2.2. OFDR Signals of SMF-28 and ZrO_2_-Doped Fibers at Room Temperature before Heat Treatment

**Figure 2:**
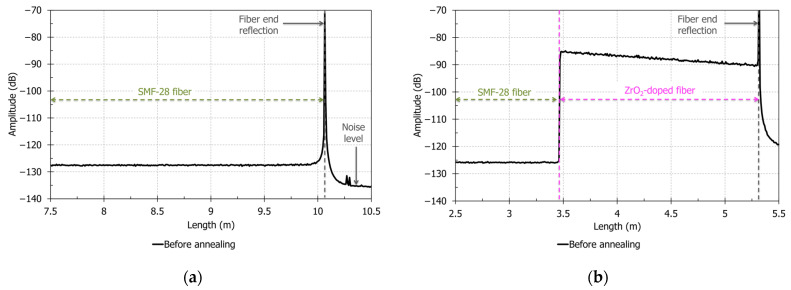
Rayleigh backscattering signal amplitude with OFDR measurement at room temperature before annealing (**a**) for SMF-28 fiber; (**b**) for the ZrO_2_-doped fiber.

A pristine SMF-28 fiber is used to compare the performance of the ZrO_2_-doped fiber for temperature sensing. A 1.8-m long ZrO_2_-doped fiber is spliced to another SMF-28 fiber to observe the amplitude improvement of the ZrO_2_-doped fiber. SMF-28 and spliced ZrO_2_-doped fibers are successively connected to the OFDR interrogator to make measurements at room temperature (~22 °C). Figure 2a,b shows the OFDR signal amplitudes of SMF-28 and ZrO_2_-doped fibers before any heat treatments, respectively.

Refractive index values of both fibers and their OFDR signal characteristics are indicated in Table 1. SMF-28 fiber has a low backscattering signal: in Figure 2a, the signal amplitude is around −128.0 dB, while the device noise level is around −135.8 dB. SMF-28 fiber does not show visible losses by OFDR on short range: amplitude slope is around 0 dB/m between 7.7 and 9.7 m. Backscattering signal amplitude and losses on SMF-28 fiber are very low since this standard single-mode fiber is optimized for telecommunications at around 1550 nm [40,41]. In Figure 2b, the OFDR signal amplitude in the ZrO_2_-doped fiber reaches a 40.5 dB increase compared to SMF-28 fiber. With OFDR signal losses of 2.8 dB/m, the ZrO_2_-doped fiber has an improved signal on a length of 14.5 m. These characteristics may be explained by the presence of zirconia entities inside the fiber’s core. The ZrO_2_-doped fiber amplitude enhancement is at the same level than in the works of Yan et al. (~40–45 dB) with nanograting-inscribed fiber [7]. The ZrO_2_-doped fiber OFDR losses are lower than in the first works on nanograting-based distributed fiber sensor (10 dB/m), but a little higher than in the optimized studies by Wang et al. (1 dB/m) [7,12]. These good OFDR characteristics observed for the ZrO_2_-doped fiber should allow better temperature measurements on several meters compared to SMF-28 fiber.

## 3. OFDR Sensing Results of Tests at 800 °C

### 3.1. OFDR Measurement Conditions for Temperature Sensing Performance Tests

**Figure 3:**
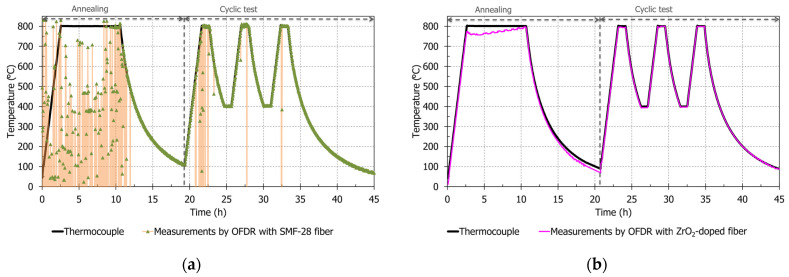
Temperature measured with SMF-28 and ZrO_2_-doped fibers during annealing and cyclic test. The OFDR reference is the last measured OFDR measurement at the ending of the cyclic test (at 22 °C). (**a**) OFDR temperature measurements from SMF-28 fiber; to have a better view of variations (due to correlation errors), green dots linked by an orange curve are plotted; (**b**) OFDR temperature measurements of the ZrO_2_-doped fiber.

SMF-28 fiber and ZrO_2_-doped fiber are tested up to 800 °C to analyze their temperature sensing performance. For heat treatments, fibers are tested in a 90 cm-long three-zone furnace having a 40 cm-long heating stable zone. A type-N thermocouple is put at the center position of the furnace to monitor temperature. The ZrO_2_-doped fiber and SMF-28 fiber are successively tested in the same furnace. Polymer fiber coatings are mechanically removed on a length of one meter before putting the fiber inside the furnace. This step permits to obtain bare fiber and therefore removes all incertitude or influence of coating material on OFDR measurements in the heating zone. Bare fibers are inserted inside two different steel capillaries to have easier fiber handling. Each fiber with its steel capillary is tested separately in the same furnace. Capillaries are not closed at their extremities, so fibers are tested in ambient atmosphere without forced air circulation. Each fiber is tested following equivalent thermal treatments, with an OFDR measurement realized every two minutes (one OFDR measurement lasts around 10 s). A first annealing step is conducted at 800 °C for 8 h to start stabilization of physical and chemical properties of fibers. After cooling, fibers are tested according to a “cyclic test” between 400 and 800 °C according to the following time steps: 1 h at 800 °C, 1 h at 400 °C, 1 h at 800 °C, 1 h at 400 °C and 1 h at 800 °C. Each heating step is conducted at a speed of 5 °C/min. The cooling step is limited in speed due to the passive furnace cooling: thus, cooling speed is under 5 °C/min. The thermal cycle is visible in Figure 3: following the black curve corresponding to values obtained with the thermocouple.

### 3.2. OFDR Temperature Measurements during Heat Treatment at 800 °C

Figure 3a,b corresponds to temperatures measured at the center of the furnace with the external thermocouple and with OFDR for one of both studied fibers. For both figures, the black curve corresponds to thermocouple’s measurements. The orange curve with green triangles (Figure 3a) and the purple curve (Figure 3b) are respectively the temperature measured by using OFDR measurements on SMF-28 and on ZrO_2_-doped fibers at furnace center position. The OFDR reference is the last measured OFDR measurement at the ending of the cyclic test at 22 °C.

**Figure 4:**
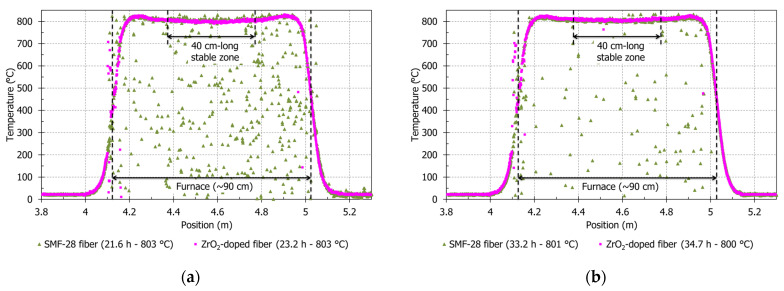
OFDR Temperature at 800 °C along annealed ZrO_2_-doped and annealed SMF-28 fibers compared to reference (last measurement of each test). The shown positions correspond to positions of ZrO_2_-doped fiber. The positions of SMF-28 fiber are subtracted from 3.992 m to put the heating zone at the same position than the one from ZrO_2_-doped fiber. The 40 cm-long stable zone corresponds to the stable heating zone of the furnace. (**a**) During the first step at 800 °C of the cyclic test. (**b**) During the third step at 800 °C of the cyclic test.

Figure 4a,b corresponds to measured temperatures along SMF-28 and ZrO_2_-doped fibers respectively during the first step and the last step of the cyclic test (measurement times are indicated in legends). These figures highlight the correlation error evolution with time.

In Figure 3a, correlation errors on SMF-28 are clearly visible during annealing (before 11.9 h and above 490 °C) and also in the first step of this cyclic test (from 20.7 h at 536 °C to 23.3 h at 628 °C): some points do not follow the thermocouple measurement curve, especially at 800 °C (measured temperatures could be under absolute zero temperature). As mentioned in the introduction, permanent changes in Rayleigh scattering profile signal and the low OFDR signal amplitude explain these correlation errors. Still at the furnace center position, results are better during the last step (between 32.3 and 33.3 h—Figure 4b), because the reference for correlation is at the end of cyclic test: thus, the OFDR signal at 800 °C is more like the reference than another OFDR signal measured at the beginning of the test. During this last step at 800 °C, in Figure 4b at 33.2 h, OFDR results on SMF-28 fiber has some positions with correct measurements (around 800 °C). In this same measurement, errors of several hundred degrees are still present, despite previous heating steps. It could be possible to improve correlation by improving the thermal annealing (a possibility as suggested by Buric et al. may be 2 h at 900 °C [9]).

In Figure 3b, the ZrO_2_-doped fiber does not show any correlation error during the cyclic test at the furnace center position at around 800 °C. This is also observed in Figure 4a along the ZrO_2_-doped fiber (at 23.2 h). However, in Figure 4b, an error is visible at 4.51 m: the measured temperature is 763.6 °C, while two OFDR gauges around this position give a temperature equal to 805.1 and 801.8 °C. We may consider this error as insignificant compared to results obtained from SMF-28 fiber. On ZrO_2_-doped fiber, OFDR temperature averages along the 40 cm stable zone in these two measurements are indicated in Table 2, which are respectively 800 ± 4 °C and 807 ± 4 °C. These values are near the thermocouple temperature value, respectively 803 ± 6 °C and 800 ± 6 °C (with standard deviation of ±0.75% on the thermocouple measurement). Moreover, standard deviations from OFDR measurements are low: under 1 %.

During the isothermal annealing step at 800 °C, OFDR temperature measured at the furnace’s center with the ZrO_2_-doped fiber decreases from 775.2 to 757.4 °C (between 2.7 and 3.2 h), before increasing up to 797.5 °C (at 10.6 h). Thus, to obtain stable Rayleigh signature along the fiber and stable OFDR results at 800 °C, annealing is required to stabilize properties of zirconia entities and silica glass. A better annealing step should allow better OFDR stability at high temperature: indeed, there are sometimes variations of several degrees (e.g., around 4 °C at 28.9 h) between two successive OFDR measurements during isothermal steps of cyclic test (temporally separated by 2 min), while thermocouple measurements do not present temperature variation between these two measurements. Additionally, OFDR results on the ZrO_2_-doped fiber show continuous temperature increase shift at 800 °C: in Table 2, averaged OFDR temperature changes from 800 ± 4 °C to 807 ± 4 °C, while the thermocouple temperatures are respectively 803 ± 6 °C and 800 ± 6 °C. Continuous shifts could be explained by physical changes (glass relaxation), germanium and phosphorus migrations or zirconia changes in size or in structure (for example: from tetragonal phase to monoclinic phase). The quick variations of a few degrees Celsius are not explained, but they could be linked to previous explanations (with phenomena at a higher “amplitude”).

### 3.3. Evolution of OFDR Signal Amplitude for Both Fibers after Heat Treatment

**Figure 5:**
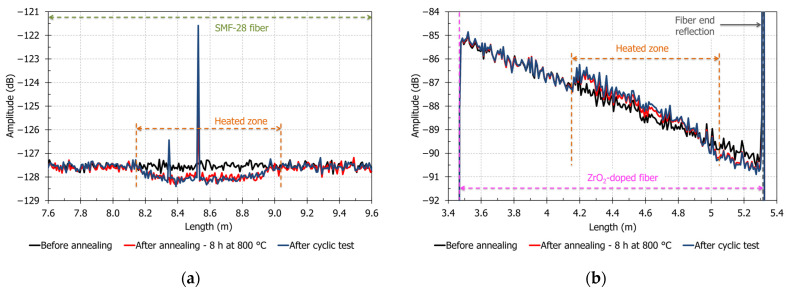
OFDR signal amplitude of tested fibers before annealing, after annealing 8 h at 800 °C and after cyclic test between 400 and 800 °C. (**a**) SMF-28 fiber; (**b**) ZrO_2_-doped fiber.

To have a better understanding of the effects of thermal treatments on the OFDR signal and temperature measurements, we study the evolution with time of backscattering signal amplitude versus position. Figure 5a,b shows three amplitude measurements versus position for SMF-28 fiber and ZrO_2_-doped fiber. The first OFDR measurement at room temperature before annealing is shown in black. The red curve is the OFDR measurement made between annealing and cyclic test (at the lowest temperature). The blue curve is the OFDR measurement at the end of the test (room temperature).

Heat treatment on SMF-28 fiber induces a permanent decrease of the OFDR signal amplitude after heat treatment in the heated zone (between 8.15 and 9.05 m—Figure 5a). In this zone, the amplitude decreases by 1 dB in average after annealing step. This phenomenon may be due to fiber glass relaxation at high temperature. For example, Sakaguchi et al. observed that optical fibers heated above 1050 °C present glass relaxation accompanied by a decrease of glass fictive temperature and a decrease of Rayleigh backscattering [42]. In Figure 5a, two intense peaks are locally observed at 8.35 m and at 8.53 m after cyclic test. These peaks correspond to local reflection, which can be explained by strains imposed by steel dust or by the steel capillary. Correlation errors for SMF-28 fiber discussed in Section 3.2 and observed in Figure 3a appear before the apparition of the OFDR signal amplitude peaks. These peaks are not responsible for poor measurement performance at high temperature for SMF-28 fiber.

The ZrO_2_-doped fiber has a different behavior: in Figure 5b, between 4.15 and 5.05 m, the heated zone shows local increases of OFDR amplitude and OFDR losses. Amplitude evolutions, calculated by linear regression, are numerically shown in Table 3. After cyclic test, compared to the pristine fiber, signal amplitude increases of 0.6 dB at the beginning of the heated zone (at around 4.15 m) and the losses increase from −2.8 dB/m to −3.2 dB/m. Currently, we can only make hypotheses to explain Rayleigh signal amplitude changes, due to characteristics of ZrO_2_-doped fiber. It is difficult to make physical or chemical characterizations of zirconia entities in the core fiber: indeed, core volume proportion is around 0.1 % and zirconia concentration in the core is also low (1.9 mol%). At this state of this study, our hypothesis is, at 800 °C, temperature is high enough to relax glass “structure” or to decrease stress induced by glass matrix around ZrO_2_ entities. The relaxation may allow slow and small physical changes of ZrO_2_ entities like crystallite size, crystal phase change (from metastable tetragonal phase to stable monoclinic phase under 1167 °C), nucleation and crystallization of nanoparticles in glass matrix or chemical diffusion. These changes of zirconia entities characteristics would increase Rayleigh scattering in glass fiber’s core. This increase of Rayleigh scattering due to particles is high enough to counterbalance the decrease of Rayleigh scattering due to relaxation of silica glass, which should occur at the same time during test at high temperature (OFDR signal amplitude of SMF-28 fiber decreases by 1 dB during the same test at 800 °C).

## 4. Conclusions

This paper presents a single-mode zirconia-doped fiber as a potential solution for temperature measurement by OFDR at 800 °C and a 40 cm long section has been tested.

Amplified Rayleigh backscattering is obtained by doping the silica fiber with zirconia by MCVD method coupled with “solution doping technique”. This approach does not require laser-inscription of nanograting, which is an additional step before annealing and use of the fiber. We suppose that the tetragonal zirconia particles initially present in preform’s core are conserved in the drawn ZrO_2_-doped fiber, due to a lower drawing temperature than melting temperature of bulk zirconia. These particles inside the optical fiber’s core induce additional Rayleigh scattering. Thus, pristine ZrO_2_-doped fiber has an amplitude increase of 40 dB compared to SMF-28 fiber and losses of 2.8 dB/m. When the OFDR reference measurement is taken at 22 °C, after fiber annealing, temperature measurements with the ZrO_2_-doped fiber show less correlation errors during successive heating steps at 800 °C than the ones from SMF-28 fiber. Moreover, the measured values during the cyclic test are in correlation with those given by the thermocouple put in the furnace. This improvement is explained by the high OFDR signal amplitude enhancement along ZrO_2_-doped fiber and by the low evolution of Rayleigh signal with time at elevated temperatures, especially after an 8 h annealing step at 800 °C. The annealing step induces an additional OFDR signal amplitude increase of 0.4 dB and OFDR losses evolve up to 3.1 dB/m along the heating zone. Nevertheless, signal stability is not completely reached with the 8 h annealing step at 800 °C: as there is a small shift over time during cyclic test. The cyclic step induces a second permanent evolution of amplitude and losses (+0.2 dB and losses of 3.2 dB/m) at room temperature. These changes in the Rayleigh scattering signal amplitude seem to be linked to simultaneous changes from zirconia entities and glass matrix, as SMF-28 fiber behavior is opposite (with amplitude decrease of 1 dB). These backscattering signal changes create a continuous temperature shift at 800 °C for the ZrO_2_-doped fiber (+7 °C between two steps of cyclic test at 800 °C). To have better temperature sensing stability at 800 °C or to use the ZrO_2_-doped fiber at higher temperatures, a complete annealing study will have to be realized to determine better annealing conditions.

Complementary studies on fiber core compositions and fiber fabrication conditions could also be conducted to improve the ZrO_2_-doped fiber’s performances for high temperature sensing: e.g., lower OFDR signal amplitude losses, better Rayleigh scattering signal stability.

## 5. Patents

A patent resulted from the work reported in this manuscript.

## Figures and Tables

**Table 1:** Characteristics of pristine SMF-28 fiber and pristine ZrO_2_-doped fiber.

	SMF-28 Fiber	ZrO_2_-Doped Fiber
Refractive index difference (at 960 nm)	5.1 × 10^−3^	8.1 × 10^−3^
n_g_ (at 1573 nm)	1.4679	1.4709
OFDR enhancement compared to SMF-28 fiber ^1^ (dB)	Undefined	40.5
OFDR losses (dB/m)	~0	−2.8
Enhanced fiber length (m)	Undefined	14.5

^1^ OFDR enhancement value does not consider the splicing losses between SMF-28 and ZrO_2_-doped fibers (equal to 1.3 dB in transmission according to the measurement by cut-back technique).

**Table 2:** OFDR temperature averages and standard deviations on the ZrO_2_-doped fiber in the furnace 40 cm-long stable zone at two different times during cyclic test (last measurement at 22 °C is chosen as the reference for both cases).

ZrO_2_-Doped Fiber	First Cyclic Test Step at 23.2 h 803 ± 6 °C		Last Cyclic-Test Step at 34.7 h 800 ± 6 °C	
Calculations along the Furnace Stable Zone	Average	Standard Deviation	Average	Standard Deviation
Temperature measured by OFDR (°C)	800	4	807	4

**Table 3:** Evolution of the OFDR signal amplitude of the ZrO_2_-doped fiber before annealing, after annealing 8 h at 800 °C and after cyclic test between 400 and 800 °C.

ZrO_2_-Doped Fiber Characteristics	Before Heat Treatment	After Heat Treatment	
		After Annealing	After Cyclic Test
OFDR amplitude enhancement compared to SMF-28 fiber (dB)	40.5	40.9	41.1
OFDR amplitude increase induced by heat treatment (dB)	Undefined	0.4	0.6
OFDR amplitude losses (dB/m)	2.8	3.1	3.2

## Appendix A

Group index at the middle of tested OFDR wavelength range for each tested optical fiber is necessary for OBR software to process OFDR data from as a function of time to as a function of a distance.

We start by calculating refractive index of silica glass cladding at wavelength *λ* = 1573 nm (which is equal to 1.4679) with Sellmeier formula (Equation (A1)) and coefficients determined by Malitson (Table A1) [43]:

(A1) $${\left(n_{silica\;glass}\left(\lambda \right)\right)}^{2}-1=\frac{B_{1}\times \lambda^{2}}{\lambda^{2}-C_{1}}+\frac{B_{2}\times \lambda^{2}}{\lambda^{2}-C_{2}}+\frac{B_{3}\times \lambda^{2}}{\lambda^{2}-C_{3}}$$

with *λ* in micrometer, *B*_1_, *B*_2_, *B*_3_, *C*_1_, *C*_2_, *C*_3_ are coefficients of Sellmeier’s equation defined in Table A1 for pure silica glass.

**Table A1:** Coefficients of Sellmeier’s equation for silica glass according to Malitson [43].

*B*_1_	*B*_2_	*B*_3_	*C*_1_	*C*_2_	*C*_3_
0.6961663	0.4079426	0.8974794	0.0684043²	0.1162414²	9.896161²

Group index of light in silica glass at wavelength *λ* is defined by:

(A2) $$n_{g}{}_{silica\;glass}\left(\lambda \right)=n_{silica\;glass}\left(\lambda \right)-\lambda \times \frac{dn_{silica\;glass}\left(\lambda \right)}{d\lambda }$$

First derivative of refractive index of silica glass, $\frac{dn_{silica\;glass}\left(\lambda \right)}{d\lambda }$, is defined by:

(A3) $$\frac{dn_{silica\;glass}\left(\lambda \right)}{d\lambda }=-\lambda \times \frac{\frac{B_{1}\times C_{1}}{{\left(\lambda^{2}-C_{1}\right)}^{2}}+\frac{B_{2}\times C_{2}}{{\left(\lambda^{2}-C_{2}\right)}^{2}}+\frac{B_{3}\times C_{3}}{{\left(\lambda^{2}-C_{3}\right)}^{2}}}{n_{silica\;glass}\left(\lambda \right)}$$

Group index of silica glass at 1573 nm is equal to 1.4628.

Brückner demonstrated that refractive index of germanium or phosphorus-doped silica glasses are respectively proportional to molar concentration of germanium oxide (*d_GeO_*_2_) or phosphorus oxide (*d_P_*_2*O*5_) [38]:

(A4) $$n_{GeO_{2}-doped\;silica\;glass}\left(\lambda \right)=n_{silica\;glass}\left(\lambda \right)+d_{GeO_{2}}\times 1.4145\times 10^{-3}$$

(A5) $$n_{P_{2}O_{5}-doped\;silica\;glass}\left(\lambda \right)=n_{silica\;glass}\left(\lambda \right)+d_{P_{2}O_{5}}\times 1.652\times 10^{-3}$$

*d_GeO_*_2_ and *d_P_*_2*O*5_ do not change with wavelength. Thus, derivative of refractive index of germanium or phosphorus-doped silica glass are equal to the one of silica glass. So, group index of germanium or phosphorus-doped silica glass fiber can be defined by:

(A6) $$n_{g}{}_{GeO_{2}-doped\;silica\;glass}\left(\lambda \right)=n_{g}{}_{silica\;glass}\left(\lambda \right)+∆n_{core/cladding}$$

(A7) $$n_{g}{}_{P_{2}O_{5}-doped\;silica\;glass}\left(\lambda \right)=n_{g}{}_{silica\;glass}\left(\lambda \right)+∆n_{core/cladding}$$

with $∆n_{core/cladding}$ is refractive index difference between core and cladding of studied optical fiber.

It is obvious Equation (A6) can be used for SMF-28 fiber which is doped by germanium. We consider that Equation (A6) can also be used for studied ZrO_2_-doped fiber as zirconia, phosphorus and germanium concentrations are low in core’s fiber. As a reminder, measured core/cladding refractive index differences for SMF-28 and ZrO_2_-doped fibers are respectively 5.1 × 10^−3^ and 8.1 × 10^−3^. For SMF-28 and ZrO_2_-doped fibers, calculated group delays at 1573 nm are respectively 1.4679 and 1.4709.
